# Additional ORFs in Plant LTR-Retrotransposons

**DOI:** 10.3389/fpls.2020.00555

**Published:** 2020-05-26

**Authors:** Carlos M. Vicient, Josep M. Casacuberta

**Affiliations:** Structure and Evolution of Plant Genomes Group, Centre for Research in Agricultural Genomics, CSIC-IRTA-UAB-UB, Campus UAB, Edifici CRAG, Barcelona, Spain

**Keywords:** additional ORF, antisense, env, LTR-retrotransposon, retrovirus

## Abstract

LTR-retrotransposons share a common genomic organization in which the 5′ long terminal repeat (LTR) is followed by the *gag* and *pol* genes and terminates with the 3′ LTR. Although GAG-POL-encoded proteins are considered sufficient to accomplish the LTR-retrotransposon transposition, a number of elements carrying additional open reading frames (aORF) have been described. In some cases, the presence of an aORF can be explained by a phenomenon similar to retrovirus gene transduction, but in these cases the aORFs are present in only one or a few copies. On the contrary, many elements contain aORFs, or derivatives, in all or most of their copies. These aORFs are more frequently located between *pol* and 3′ LTR, and they could be in sense or antisense orientation with respect to *gag*-*pol*. Sense aORFs include those encoding for ENV-like proteins, so called because they have some structural and functional similarities with retroviral ENV proteins. Antisense aORFs between *pol* and 3′ LTR are also relatively frequent and, for example, are present in some characterized LTR-retrotransposon families like maize Grande, rice RIRE2, or *Silene* Retand, although their possible roles have been not yet determined. Here, we discuss the current knowledge about these sense and antisense aORFs in plant LTR-retrotransposons, suggesting their possible origins, evolutionary relevance, and function.

## Introduction

**FIGURE 1:**
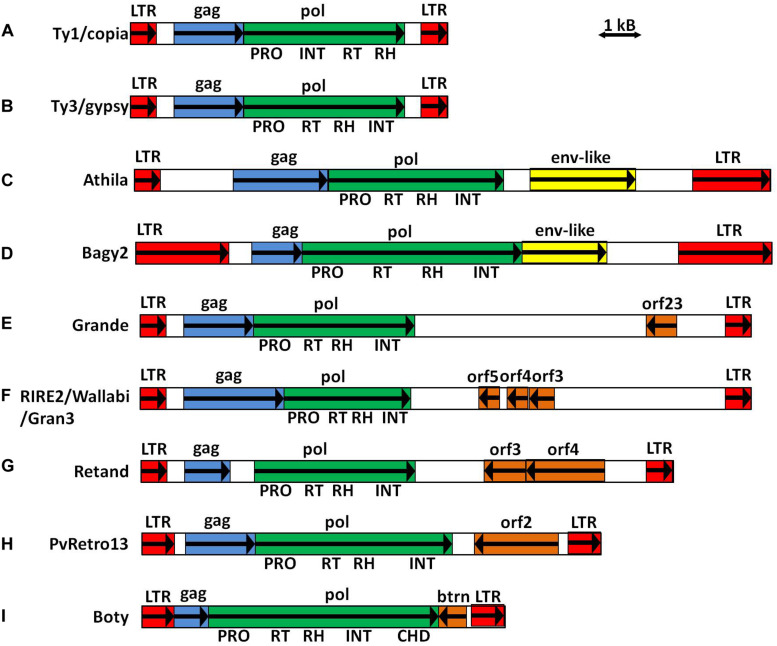
Schematic representation of LTR-retrotransposons. **(A)** Model Ty1/copia element. **(B)** Model Ty3/gypsy element. **(C)** *Arabidopis thaliana* Athila (Wright and Voytas, 2002). **(D)** Barley Bagy2 (Vicient et al., 2001). **(E)** Maize Grande (Gómez-Orte et al., 2013). **(F)** Rice RIRE2 (Ohtsubo et al., 1999), Wallabi and Gran3 (Wicker et al., 2007). **(G)** *Silene latifolia* Retand (Kejnovsky et al., 2006). **(H)** *Phaseolus vulgaris* PvRetro13 (Gao et al., 2014). **(I)** *Botrytis cinerea* Boty (Zhao et al., 2011).

LTR-retrotransposons are transposable elements (TEs) characterized by the presence of two long direct repeats (long terminal repeats, LTRs) flanking an internal region that contains the *gag* and *pol* genes encoding proteins required for transposition (Figures 1A,B). Long terminal repeats provide the promoters and terminators associated with the transcription of the LTR-retrotransposon by RNA polymerase II (Kumar and Bennetzen, 1999). The internal region contains the primer binding site (PBS) and the polypurine tract (PPT), both used during the retrotransposition process. The PBS is a 10–20-nucleotide sequence located next to the 5′ LTR that can partly base-pair with the 3′ end of a cytoplasmic tRNA. The PBS is used to prime the synthesis of the first DNA strand during the retrotranscription process. The PPT is a short stretch of purine-rich DNA (8–49 nt) located in the internal region next to the 3′ LTR and is used to prime the synthesis of the second DNA strand during retrotranscription. The internal region also contains the *gag* and *pol* genes, which encode all the proteins necessary for the retrotranscription and integration processes not provided by the cell. *Gag* encodes the structural proteins, including capsid (CA) and nucleocapsid (NC), that assemble into virus-like particles (VLPs) Dodonova et al., 2019). *Pol* encodes the proteins that provide the enzymatic machinery for reverse transcription and integration into the host genome: aspartic proteinase (AP), reverse transcriptase (RT), RNase H (RH), and integrase (INT) Kumar and Bennetzen, 1999).

As sequence data accumulate, the recognizing of sequences encoding for additional proteins (aORFs) in the internal region of plant LTR-retrotransposons seems to be more frequent (Neumann et al., 2019). The aORF can be found in LTR-retrotransposon families with low or high copy numbers, in sense or antisense orientation with respect to the *gag*-*pol* genes, and upstream or downstream of them (Steinbauerová et al., 2011). Among them, those located between *pol* and 3′ LTR in sense or in antisense with respect to the *gag* and *pol* are the most frequent.

## Plant LTR-Retrotransposons with Conserved aORFs in Sense Orientation Between *pol* and 3′ LTR

Retroviruses and LTR-retrotransposons share many structural features, and the main difference is that all retroviruses contain a third coding domain in their internal region called *env* that is located between *pol* and 3′ LTR. *Env* encodes for proteins involved in interacting with cellular receptors and mediate fusion of the host and viral membranes (Nisole and Saïb, 2004). Since retrotransposons have an intracellular retrotransposition cycle, it was initially thought that they do not need the ENV protein and, in fact, most do not have it. However, the presence of coding domains in addition to the *gag* and *pol* genes between *pol* and 3′ LTR in the same sense as *gag* and *pol* (aORFs-3S) have been described in some LTR-retrotransposons in insects (Song et al., 1994) and in plants (Vicient et al., 2001; Wright and Voytas, 2002; Laten and Gaston, 2012). They encode proteins with certain similarities to ENV, suggesting that they may also exist in, at least, some LTR-retrotransposons (Figures 1C,D).

One of the conserved characteristics of the retroviral *env* domains is that they code for proteins with transmembrane domains, a characteristic that some of these aORFs-3S also have, which may suggest some functional similarity (Vicient et al., 2001). On the other hand, the retroviral mRNA is usually spliced to give rise to a message capable of expressing the envelope protein. Similar splicing events have been reported for some plant retrotransposons like barley Bagy2 (Vicient et al., 2001). Together, the data suggest that aORFs-3S could encode proteins with functional similarities to retroviral ENV and therefore they are often called *env-like* domains (Carvalho et al., 2010). However, an ENV-like function in plants remains controversial because the plant cell wall may represent a barrier to the interaction of ENV with cellular receptors present in the plasma membrane. As a consequence, it has been proposed that ENV-like proteins in plants may have a different function than in retroviruses as, for example, to modify the molecular size exclusion limit of plasmodesmata (Carrington et al., 1996) or to serve as chaperone proteins to facilitate replication (Havecker et al., 2004).

## Plant LTR-Retrotransposons with Conserved aORFs in Antisense Orientation Between *pol* and 3′ LTR

The presence of coding domains in addition to the *gag* and *pol* genes between *pol* and 3′ LTR in antisense with respect to *gag* and *pol* (aORFs-3R) has been described in different plant LTR-retrotransposons. Some examples are maize Grande1, rice RIRE2, Retand from *Silene latifolia*, and PvRetro13 from *Phaseolus vulgaris* (Martínez-Izquierdo et al., 1997; Ohtsubo et al., 1999; Kejnovsky et al., 2006; Figures 1E–H). They are also present in non-plant species as, for example, in Boty from *Botrytis cinerea* and *Sclerotinia sclerotiorum* (Zhao et al., 2011; Figure 1I). The function of these aORF-3Rs is not known, but their presence in most of the copies of a family, with a degree of sequence conservation similar to that of other retrotransposon-encoded proteins, suggests that they may be important for the retrotransposition process (Ohtsubo et al., 1999; Gómez-Orte et al., 2013).

Although a complete analysis of the presence, species distribution, and types of these aORF-3Rs is not yet available, the current data indicates that some of them are distributed in several species. For example, the sequences of the aORF-3R of RIRE2, Wallabi, and Gran3, from different species of the genus *Oryza*, show similarities with the aORF-3R of Grande from *Zea* species (Ohtsubo et al., 1999). Retand aORF-3Rs from *S. latifolia* show homology with sequences from other species, and sequences similar to PvRetro13 aORF-3R were detected in other species (Gao et al., 2014). These homologies indicate an ancient origin, at least for some of the aORF-3R.

## Origin of Antisense aORFs

Retroviruses have the potential to capture complete or parts of cellular genes in a process known as gene transduction. Gene transduction events have also been described in some Class I TEs. For example, human L1 retrotransposons can capture gene fragments by transduction (Goodier et al., 2000). There are also some described examples in plants. Bs1, a maize LTR-retrotransposon, has transduced sequences from different host genes (Elrouby and Bureau, 2010). A total of 400 genes have been identified as transposon-captured genes in maize (Schnable et al., 2009), 672 in rice and 1343 in sorghum (Jiang and Ramachandran, 2013). In maize, the majority of TE-captured genes were from Helitron elements, in rice from Pack-MULEs, and in sorghum from LTR-retrotransposons, and a high percentage of LTR-retrotransposon-captured genes are still functional in sorghum (Jiang and Ramachandran, 2013). However, retrotransposon-transduced gene sequences are usually in the same sense as the retrotransposon *gag* and *pol* genes. Moreover, the lack of gene sequences similar to that of aORFs outside the TEs suggests that aORFs are not transduced gene sequences. Another possible origin of the aORF is the insertion of a TE that, once inserted, became part of the element, losing part of its structure. However, although nested insertions of TEs are relatively frequent in plant genomes (SanMiguel and Bennetzen, 1998), the lack of similarity of the aORF sequences with that of other TEs or viruses does not support this hypothesis. In consequence, the origin of most of these aORFs remains unknown.

## Function of Antisense aORFs

No clear similarities with other proteins in databases have been described for any of the proteins encoded by aORFs-3R. However, some of these peptides localize in the nucleus, as the one encoded by Grande (GENE23; Gómez-Orte et al., 2013), and the one encoded by PvRetro13 (ORF2) contains a conserved SMC domain (structural maintenance of chromosomes) that binds DNA and acts in organizing and segregating chromosomes for partitioning (Marchler-Bauer et al., 2011). These results suggest that these proteins may fulfill some nuclear function. Moreover, the aORF-3R protein encoded by Retand (ORF4) contains a transposase 28 domain (pfam04195), suggesting a possible role in the retrotransposition process. During retrotransposition, the pre-integration complex (PIC) produced in the cytoplasm must translocate to the nucleus (McLane et al., 2008). In some retrotransposons, like the fission yeast Tf1, the nuclear localization signal is provided by GAG (Kim et al., 2005), but in retroviruses some accessory proteins are involved (Vogt, 1997; Kogan and Rappaport, 2011). Many retroviruses encode more ORFs in addition to *gag*, *pol*, and *env*, called accessory factors. These ORFs could be in antisense orientation and be located between *pol* and 3′ LTR. The Accessory factors encode for structural and enzymatic proteins essential for the regulation of transcription (Tat), the transport of unspliced and partially spliced viral RNAs from the nucleus into the cytoplasm (Rev), and others (Vif, Vpr, Vpu, Vpx, and Nef) (Sauter and Kirchhoff, 2018). All these suggest that the proteins encoded by aORFs-3R in LTR-retrotransposons may play a role similar to some of the retroviral accessory proteins, regulating the retrotransposition process.

## Transcription of aORFs-3R

The transcription of *gag*-, *pol*-, and *env*-like genes in LTR-retrotransposons is directed by a promoter located in the 5′ LTR. In maize Grande, the region corresponding to *gene23* (aORF-3R) is ubiquitously transcribed in a relatively high level in antisense with respect to the *gag*-*pol* genes. This transcription is directed by a promoter located in the upstream region of *gene23* (Gómez et al., 2006; Vicient, 2010; Gómez-Orte et al., 2013). A weak ubiquitous expression was also detected for Retand aORFs-3R (Kejnovsky et al., 2006). Antisense promoters are not unusual in retrovirus. For example, HTLV has an antisense promoter located inside the 3′ LTR (Barbeau and Mesnard, 2011). Antisense promoters have been also identified in other retrotransposons as, for example, the apple Mdoryco1-1 (Wang et al., 2017), *Arabidopsis thaliana* AtRE1 (Kato et al., 2005), and *Drosophila hydei* micropia (Lankenau et al., 1994). However, in the case of micropia, it transcribes two antisense RNAs of 1.0 and 1.6 kb with no protein coding capacity. Antisense non-coding transcripts have also been described for the Drosophila non-LTR TART element (Danilevskaya et al., 1999) and in mouse IAP elements that have both sense and antisense promoter activities in their LTRs (Druker et al., 2004). In view of these last examples, we cannot rule out that, at least in some cases, the production of antisense mRNAs has itself some regulatory roles. Antisense transcripts may produce double-stranded RNAs (dsRNAs) when they hybridize with the genomic RNA of the LTR-retrotransposon, generating 21–24-nt siRNAs, which can act as inhibitors of the retrotransposition by a dsRNA-mediated silencing mechanism. These two possible functions based on the generation of a protein or on the generation of dsRNA can be true simultaneously and represent a fine regulation of the LTR-retrotransposition mechanism.

## Conclusion

Additional open reading frames located between the *pol* gene and the 3′ LTR are present in some plant LTR-retrotransposon families. Sense aORFs show some functional and structural characteristics similar to the *env* genes in retroviruses, although their possible roles in retrotransposition remain unclear. Antisense aORFs are also present in different retrotransposon families, but their functions are yet unknown. The nuclear localization identified in some cases and the comparison with the antisense genes of retroviruses suggest they may play a regulatory role in retrotransposition. Antisense transcription may also play a regulatory role itself, through a dsRNA-mediated silencing mechanism. In conclusion, we believe that it is necessary to pay more attention to the presence of this type of additional ORFs in the annotations of the TEs. We also think that it is necessary to look at the possible presence of antisense and spliced transcripts. Finally, we think it would be interesting to carry out research efforts on the possible functions that the transcripts and the proteins they encode could perform.

## Author Contributions

CV drafted the manuscript with contributions of JC. Both CV and JC revised and approved the manuscript.

## Conflict of Interest

The authors declare that the research was conducted in the absence of any commercial or financial relationships that could be construed as a potential conflict of interest.

## References

[B1] Barbeau B. Mesnard J. M. (2011). Making sense out of antisense transcription in human T-cell lymphotropic viruses (HTLVs). *Viruses* 3 456–468. 10.3390/v3050456 21994742 PMC3185765

[B2] Carrington J. C. Kasschau K. D. Mahajan S. K. Schaad M. C. (1996). Cell-to-cell and long-distance transport of viruses in plants. *Plant Cell* 8 1669–1681. 10.1105/tpc.8.10.1669 12239357 PMC161306

[B3] Carvalho M. Ribeiro T. Viegas W. Morais-Cecilio L. Rocheta M. (2010). Presence of env-like sequences in *Quercus suber* retrotransposons. *J. Appl. Genet.* 51 461–467. 10.1007/BF03208875 21063063

[B4] Danilevskaya O. N. Traverse K. L. Hogan N. C. DeBaryshe P. G. Pardue M. L. (1999). The two *Drosophila* telomeric transposable elements have very different patterns of transcription. *Mol. Cell Biol.* 19 873–881. 10.1128/mcb.19.1.873 9858610 PMC83944

[B5] Dodonova S. O. Prinz S. Bilanchone V. Sandmeyer S. Briggs J. A. G. (2019). Structure of the Ty3/Gypsy retrotransposon capsid and the evolution of retroviruses. *Proc. Natl. Acad. Sci. U.S.A.* 116 10048–10057. 10.1073/pnas.1900931116 31036670 PMC6525542

[B6] Druker R. Bruxner T. J. Lehrbach N. J. Whitelaw E. (2004). Complex patterns of transcription at the insertion site of a retrotransposon in the mouse. *Nucleic Acids Res.* 32 5800–5808. 10.1093/nar/gkh914 15520464 PMC528799

[B7] Elrouby N. Bureau T. E. (2010). Bs1, a new chimeric gene formed by retrotransposon-mediated exon shuffling in maize. *Plant Physiol.* 153 1413–1424. 10.1104/pp.110.157420 20488894 PMC2899935

[B8] Gao D. Abernathy B. Rohksar D. Schmutz J. Jackson S. A. (2014). Annotation and sequence diversity of transposable elements in common bean (*Phaseolus vulgaris*). *Front. Plant Sci.* 11:339. 10.3389/fpls.2014.00339 25071814 PMC4093653

[B9] Gómez E. Schulman A. H. Martínez-Izquierdo J. A. Vicient C. M. (2006). Integrase diversity and transcription of the maize retrotransposon Grande. *Genome* 49 558–562. 10.1139/g05-129 16767181

[B10] Gómez-Orte E. Vicient C. M. Martínez-Izquierdo J. A. (2013). Grande retrotransposons contain an accessory gene in the unusually long 3′-internal region that encodes a nuclear protein transcribed from its own promoter. *Plant Mol. Biol.* 81 541–551. 10.1007/s11103-013-0019-2 23423698

[B11] Goodier J. L. Ostertag E. M. Kazazian H. H. Jr. (2000). Transduction of 3′-flanking sequences is common in L1 retrotransposition. *Hum. Mol. Genet.* 9 653–657. 10.1093/hmg/9.4.653 10699189

[B12] Havecker E. R. Gao X. Voytas D. F. (2004). The diversity of LTR retrotransposons. *Genome Biol.* 5:225. 10.1186/gb-2004-5-6-225 15186483 PMC463057

[B13] Jiang S. Y. Ramachandran S. (2013). Genome-wide survey and comparative analysis of LTR retrotransposons and their captured genes in rice and sorghum. *PLoS One* 8:e71118. 10.1371/journal.pone.0071118 23923055 PMC3726574

[B14] Kato A. Endo M. Kato H. Saito T. (2005). The antisense promoter of AtRE1, a retrotransposon in *Arabidopsis thaliana*, is activated in pollens and calluses. *Plant Sci.* 168 981–986.

[B15] Kejnovsky E. Kubat Z. Macas J. Hobza R. Mracek J. Vyskot B. (2006). Retand: a novel family of gypsy-like retrotransposons harboring an amplified tandem repeat. *Mol. Genet. Genomics.* 276 254–263. 10.1007/s00438-006-0140-x 16826419

[B16] Kim M. K. Claiborn K. C. Levin H. L. (2005). The long terminal repeat-containing retrotransposon Tf1 possesses amino acids in gag that regulate nuclear localization and particle formation. *J. Virol.* 79 9540–9555. 10.1128/JVI.79.15.9540-9555.2005 16014916 PMC1181613

[B17] Kogan M. Rappaport J. (2011). HIV-1 accessory protein Vpr: relevance in the pathogenesis of HIV and potential for therapeutic intervention. *Retrovirology* 8:25. 10.1186/1742-4690-8-25 21489275 PMC3090340

[B18] Kumar A. Bennetzen J. L. (1999). Plant retrotransposons. *Annu. Rev. Genet.* 33 479–532. 10690416 10.1146/annurev.genet.33.1.479

[B19] Lankenau S. Corces V. G. Lankenau D. H. (1994). The *Drosophila* micropia retrotransposon encodes a testis-specific antisense RNA complementary to reverse transcriptase. *Mol. Cel. Biol.* 14 1764–1775. 10.1128/mcb.14.3.1764 7509447 PMC358534

[B20] Laten H. M. Gaston G. D. (2012). “Plant Endogenous Retroviruses? A Case of Mysteriuos ORFs,” in *Plant Transposable Elements. 2012. Impact on Genome Structure and Function, Topics in Current Genetics 24*, eds Grandbastien M. A. Casacuberta J. M. Berlin: Springer, 89–112.

[B21] Marchler-Bauer A. Lu S. Anderson J. B. Chitsaz F. Derbyshire M. K. DeWeese-Scott C. (2011). CDD: a conserved domain database for the functional annotation of proteins. *Nucleic Acids Res.* 39 D225–D229. 10.1093/nar/gkq1189 21109532 PMC3013737

[B22] Martínez-Izquierdo J. A. Garcia-Martinez J. Vicient C. M. (1997). What makes Grande1 retrotransposon different? *Genetica* 100 15–28. 9440255

[B23] McLane L. M. Pulliam K. F. Devine S. E. Corbett A. H. (2008). The Ty1 integrase protein can exploit the classical nuclear protein import machinery for entry into the nucleus. *Nucleic Acids Res.* 36 4317–4326. 10.1093/nar/gkn383 18586821 PMC2490736

[B24] Neumann P. Novák P. Hoštáková N. Macas J. (2019). Systematic survey of plant LTR-retrotransposons elucidates phylogenetic relationships of their polyprotein domains and provides a reference for element classification. *Mob. DNA.* 10:1. 10.1186/s13100-018-0144-1 30622655 PMC6317226

[B25] Nisole S. Saïb A. (2004). Early steps of retrovirus replicative cycle. *Retrovirology* 1 9–20. 10.1186/1742-4690-1-9 15169567 PMC421752

[B26] Ohtsubo H. Kumekawa N. Ohtsubo E. (1999). RIRE2, a novel gypsy-type retrotransposon from rice. *Genes Genet. Syst.* 74 83–91. 10.1266/ggs.74.83 10586517

[B27] SanMiguel P. Bennetzen J. L. (1998). Evidence that a recent increase in maize genome size was caused by the massive amplification of intergene retrotransposons. *Ann. Bot.* 82 37–44.

[B28] Sauter D. Kirchhoff F. (2018). Multilayered and versatile inhibition of cellular antiviral factors by HIV and SIV accessory proteins. *Cytokine Growth Factor Rev.* 40 3–12. 10.1016/j.cytogfr.2018.02.005 29526437

[B29] Schnable P. S. Ware D. Fulton R. S. Stein J. C. Wei F. Pasternak S. (2009). The B73 maize genome: complexity, diversity, and dynamics. *Science* 326 1112–1115. 10.1126/science.1178534 19965430

[B30] Song S. U. Gerasimova T. Kurkulos M. Boeke J. D. Corces V. G. (1994). An env-like protein encoded by a *Drosophila* retroelement: evidence that gypsy is an infectious retrovirus. *Genes Dev.* 8 2046–2057. 10.1101/gad.8.17.2046 7958877

[B31] Steinbauerová V. Neumann P. Novák P. Macas J. (2011). A widespread occurrence of extra open reading frames in plant Ty3/gypsy retrotransposons. *Genetica* 139 1543–1555. 10.1007/s10709-012-9654-9 22544262

[B32] Vicient C. M. (2010). Transcriptional activity of transposable elements in maize. *BMC Genomics* 11:601. 10.1186/1471-2164-11-601 20973992 PMC3091746

[B33] Vicient C. M. Kalendar R. Schulman A. H. (2001). Envelope-containing retrovirus-like elements are widespread, transcribed and spliced, and insertionally polymorphic in plants. *Genome Res.* 11 2041–2049. 10.1101/gr.193301 11731494 PMC311225

[B34] Vogt P. K. (1997). “Retroviral virions and genomes,” in *Retroviruses*, eds Coffin J. M. Hughes S. M. Varmus H. E. (Cold Spring Harbor, NY: Cold Spring Harbor Laboratory Press), 27–69. 21433348

[B35] Wang L. He Y. Qiu H. Guo J. Han M. Zhou J. (2017). Mdoryco1-1, a bidirectionally transcriptional Ty1-copia retrotransposon from *Malus*×*domestica*. *Sci. Hortic.* 220 283–290.

[B36] Wicker T. Sabot F. Hua-Van A. Bennetzen J. L. Capy P. Chalhoub B. (2007). A unified classification system for eukaryotic transposable elements. *Nat. Rev. Genet.* 8 973–982. 10.1038/nrg2165 17984973

[B37] Wright D. A. Voytas D. F. (2002). Athila4 of *Arabidopsis* and Calypso of soybean define a lineage of endogenous plant retroviruses. *Genome Res.* 12 122–131. 10.1101/gr.196001 11779837 PMC155253

[B38] Zhao M. Zhou J. Y. Li Z. D. Song W. W. Gong T. Tan H. (2011). Boty-like retrotransposons in the filamentous fungus *Botrytis cinerea* contain the additional antisense gene *brtn*. *Virology* 417 248–252. 10.1016/j.virol.2011.06.020 21802103

